# A Rare Adrenal Mass in a 3-Month-Old: A Case Report and Literature Review

**DOI:** 10.1155/2017/4542321

**Published:** 2017-02-23

**Authors:** Ashish Garg, Elza Pollak-Christian, Navneetha Unnikrishnan

**Affiliations:** SUNY Downstate Medical Center, Brooklyn, NY, USA

## Abstract

A three-month-old female infant presented with abdominal distention for 2 months. A large palpable mass in right upper quadrant was noted on physical exam. Abdominal ultrasound revealed a large heterogeneous mass with multiple cystic components. Mass was surgically excised and pathology was consistent with mature adrenal teratoma. Teratoma is a germ cell tumor mainly found in gonadal tissues. Occurrence of adrenal gland teratoma in children is very rare with less than 10 pediatric case reports in English literature. We present a rare case of primary adrenal tumor in an infant and a review of the literature.

## 1. Introduction

Teratoma is a rare germ cell tumor (GCT) that develops from totipotent stem cells which differentiate into chaotically intermixed tissues that are foreign to the anatomic site in form which they arose. Tissue components that comprise the tumor are from all three embryonic germ layers; endoderm, mesoderm, and ectoderm [1]. Incidence of GCT is very rare and is approximately 12 cases per million persons under twenty years of age [2, 3]. While in the adult population GCTs are almost always gonadal, in the pediatric population extragonadal tumors comprise two-thirds of cases. Teratoma is the most common GCT. The most common extragonadal site for teratoma is the sacrococcygeal and presacral region [3]. Additionally, teratomas have been reported to occur in the anterior mediastinum, retroperitoneum, and cranial regions. Primary teratomas in the retroperitoneum are seen in approximately 5% of cases in infants and primary adrenal teratomas are extremely rare [4]. In English literature, less than ten cases have been reported in the pediatric population (under 21 years of age). We report a case of a 3-month-old infant with primary adrenal cystic teratoma.

## 2. Case Report

**Figure 1:**
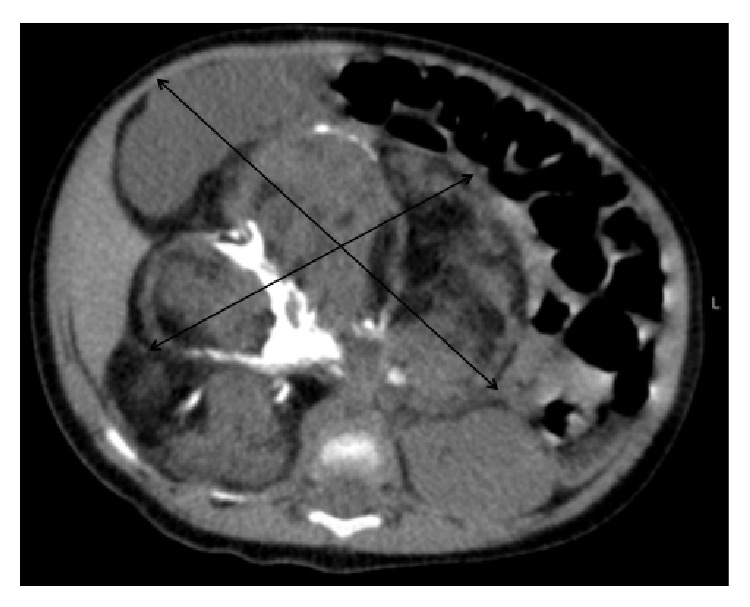
MRI of abdomen showing heterogeneous mass extending from the right suprarenal fossa and into the peritoneal cavity, measuring 10.2 cm × 10.5 cm × 8.5 cm (AP × TRV × SI). There are multiple large collections of high T2 signal consistent with cystic component degeneration.

A three-month-old female infant, without any significant past medical or perinatal history, presented with a 2-month history of a gradually increasing abdominal distention. Patient was otherwise growing and developing well. A large palpable mass was found on examination which was predominantly located in right upper quadrant, cystic in consistency. An abdominal ultrasound was performed and revealed a large heterogeneous mass (8 cm × 10 cm) with multiple cystic components and calcification with inferior displacement of right kidney. An abdominal magnetic resonance imaging revealed a heterogeneous mass extending from the right suprarenal fossa and into the peritoneal cavity measuring 10.2 cm × 10.5 cm × 8.5 cm with multiple large collections consistent with cystic components, displacing the right kidney inferiorly (Figure 1).

To rule out neuroblastoma MIBG scan was performed and was negative and urine vanillylmandelic acids (VMA) were also negative. Additionally other tumor markers, alpha-fetoprotein (AFP), human chorionic gonadotropin (hCG), and LDH, were appropriate for age. Complete blood counts were also normal including hemoglobin and hematocrit.

**Figure 2:**
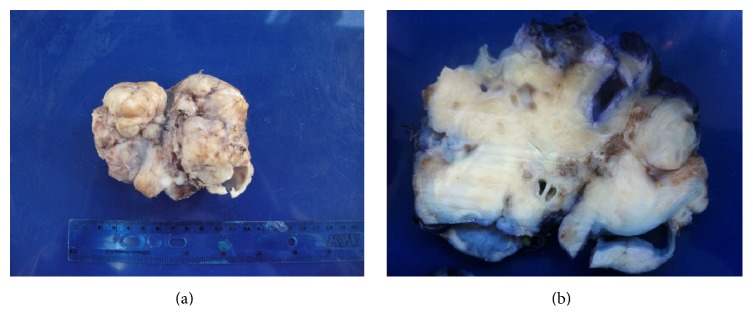
Gross appearance showing irregular tan-white mass (a) and (b) cut section showing a heterogeneous surface with solid and cystic areas along with bony areas.

**Figure 3:**
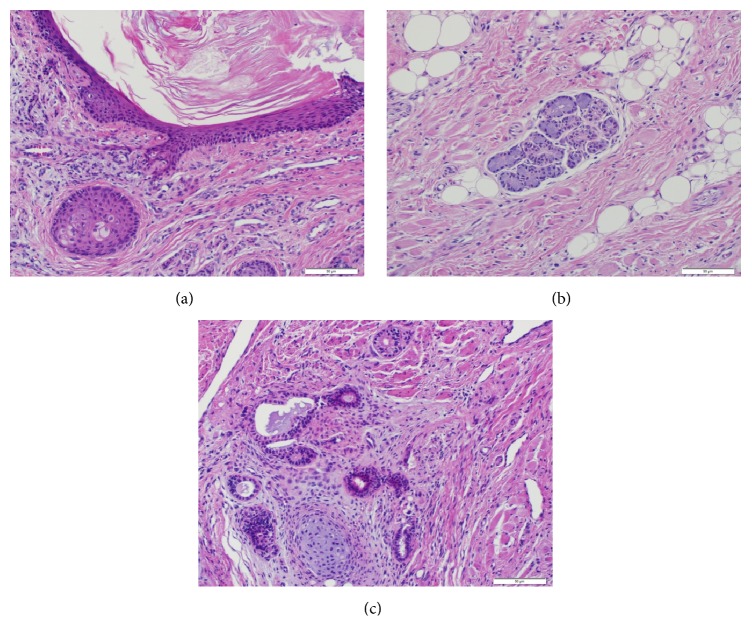
(a) Microscopic images showing cystic spaces lined by keratinized squamous epithelium; (b) skeletal muscle and salivary type glandular epithelium; (c) skeletal muscle, well differentiated cartilage, and epithelium lined glandular spaces.

The patient underwent open laparotomy. The mass was located in the right retroperitoneum pushing right colon, duodenum, IVC, portal vein, pancreas, and portal triad anteromedially and liver and gall bladder superiorly. No metastatic disease was identified. The mass was completely excised. Gross dimensions of the tumor were 10 cm × 10 cm × 8 cm, weighing 380 grams. The mass had heterogeneous quality with multiple cystic areas as well as hard bony areas. It consists of an unoriented, irregular, partially opened tan-white mass. Cut section of the mass revealed cystic spaces and solid areas. Cystic spaces were filled with clear fluid and lined with smooth tan/pink surface with focal areas of hemorrhage (Figure 2). The tumor was mainly composed of well differentiated neural tissue, skeletal muscle and bone; cystic areas lined by squamous epithelium, salivary type glandular epithelium. Intestinal and colonic mucosa were also present without any evidence of immature elements consistent with mature adrenal teratoma (Figure 3).

## 3. Discussion

Teratomas are classified as 1 of 4 variants: mature, immature, teratoma with malignant transformation, and monodermal [11]. According to the location of tumor, teratomas can be classified into gonadal and extragonadal teratomas. Gonadal teratomas are more common in adults, mostly primary neoplasms, usually take place in gonads (testes and ovaries). Extragonadal sites account for 15% of all teratomas, and the retroperitoneum in the first 6 months of life is the least common location [12]. Retroperitoneal mature cystic teratomas are characterized by a bimodal peak in incidence, occurring in the first 6 months of life and then in early childhood. Approximately half of all cases are diagnosed within the first year of life. Only 10% to 20% occur in adults over the age of 30 years [13].

Adrenal teratomas are usually benign, have no clinical manifestations, and are not associated with elevated markers unless malignancy is present. The retroperitoneum provides a large space for the mass to grow; thus tumors are typically large at the time of presentation. Patients can present with abdominal distension, abdominal pain, low back pain, and intestinal obstruction caused by compression or may be asymptomatic and the mass can be discovered incidentally. Cystic teratomas may rupture and cause sudden onset of abdominal pain, ascites, and peritonitis [1, 14].

The diagnosis of adrenal teratoma relies predominantly on imaging because the findings from laboratory examinations will often be normal. Plain abdominal film often shows calcification. Sonography can identify the cystic, solid, or complex components of the tumor [15]. Magnetic resonance imaging is better than sonography and CT to demonstrate anatomical relationships [16]. A postoperative pathologic examination is required for a definitive diagnosis. Surgical complete resection and close follow-up are recommended therapy for mature teratomas. The prognosis is favorable for benign adrenal teratoma after complete resection. In review of English literature so far 7 cases of adrenal teratoma have been reported in pediatric patients under 21 years of age and are described in Table 1.

## Figures and Tables

**Table 1:** Age, sex, presentation, location, and size of seven previously reported cases of primary adrenal teratoma in pediatric population.

Author	Age	Sex	Presentation	Side	Gross pathology	Diagnosis
Engel et al., 1968 [5]	5 yo	M	Incidental	R	Cystic 10 cm × 7.5 cm × 3.5 cm	Mature adrenal teratoma with predominant adipose component
Terada et al., 1987 [6]	18 yo	F	Incidental	R	Cystic 15 cm	Adrenal teratoma
Lam and Lo, 1999 [7]	18 yo	F	Back pain for 1 month	R	Solid 11 cm × 8 cm × 7 cm	Mature adrenal teratoma with predominant adipose component
	17 yo	M	Back pain for 1 year	L	Mixed 7.5 cm × 6 cm × 3 cm	Mature adrenal teratoma with predominant adipose component
Oguzkurt et al., 2009 [8]	45 do	M	Prenatal diagnosis of a suprarenal mass	L	Mixed 5.5 cm × 4.5 cm × 3 cm	Mature adrenal teratoma
Ersoz et al., 2011 [9]	8 yo	M	Abdominal pain	R	Cystic 10 cm × 8.5 cm × 6 cm	Neurocytoma in a mature cystic teratoma
Ciftci et al., 2013 [10]	3 mo	M	Vomiting and abdominal distension	L	Mixed 14 cm × 10 cm × 8 cm	Mature adrenal teratoma

## References

[1] Fanaroff A. A., Martin R. J., Walsh M. C. (2011). *Fanaroff and Martin's Neonatal-Perinatal Medicine, Diseases of the Fetus and Infant*.

[2] Göbel U., Schneider D. T., Calaminus G., Haas R. J., Schmidt P., Harms D. (2000). Germ-cell tumors in childhood and adolescence. *Annals of Oncology*.

[3] Kliegman R. (2011). *Nelson Textbook of Pediatrics*.

[4] Luo C.-C., Huang C.-S., Chu S.-M., Chao H.-C., Yang C.-P., Hsueh C. (2005). Retroperitoneal teratomas in infancy and childhood. *Pediatric Surgery International*.

[5] Engel R. M., Elkins R. C., Fletcher B. D. (1968). Retroperitoneal teratoma. Review of the literature and presentation of an unusual case. *Cancer*.

[6] Terada Y., Kato A., Kishi H., Umeda T., Niijima T., Yashiro N. (1987). Nuclear magnetic resonance imaging of a benign cystic teratoma in the retroperitoneum. *The Journal of Urology*.

[7] Lam K.-Y., Lo C.-Y. (1999). Teratoma in the region of adrenal gland: a unique entity masquerading as lipomatous adrenal tumor. *Surgery*.

[8] Oguzkurt P., Ince E., Temiz A., Demir S., Akabolat F., Hicsonmez A. (2009). Prenatal diagnosis of a mass in the adrenal region that proved to be a teratoma. *Journal of Pediatric Hematology/Oncology*.

[9] Ersoz S., Kucuk H., Mungan S., Turgutalp H., Imamoglu M., Kosucu P. (2011). Neurocytoma arising in an adrenal gland mature teratoma. *Fetal and Pediatric Pathology*.

[10] Ciftci I., Cihan T., Koksal Y., Ugras S., Erol C. (2013). Giant mature adrenal cystic teratoma in an infant. *Acta Informatica Medica*.

[11] Bovio S., Cataldi A., Reimondo G. (2006). Prevalence of adrenal incidentaloma in a contemporary computerized tomography series. *Journal of Endocrinological Investigation*.

[12] Bedri S., Erfanian K., Schwaitzberg S., Tischler A. S. (2002). Mature cystic teratoma involving adrenal gland. *Endocrine Pathology*.

[13] Gatcombe H. G., Assikis V., Kooby D., Johnstone P. A. S. (2004). Primary retroperitoneal teratomas: a review of the literature. *Journal of Surgical Oncology*.

[14] Pandya J. S., Pai M. V., Muchhala S. (2000). Retroperitoneal teratoma presenting as acute abdomen in an elderly person. *Indian Journal of Gastroenterology*.

[15] Wootton-Gorges S. L., Thomas K. B., Harned R. K., Wu S. R., Stein-Wexler R., Strain J. D. (2005). Giant cystic abdominal masses in children. *Pediatric Radiology*.

[16] Bellin M. F., Duron J. J., Curet P., Dion-Voirin E., Grellet J. (1991). Primary retroperitoneal teratoma in the adult: correlation of MRI features with CT and pathology. *Magnetic Resonance Imaging*.

